# Developmental Evaluation as a Tool for Spurring Age-Friendly Communities in New Jersey and Beyond

**DOI:** 10.1093/geroni/igaf122.1111

**Published:** 2025-12-31

**Authors:** Natalie Pope, Renie Carniol, Julia Stoumbos, Cathy Rowe, Emily Greenfield

**Affiliations:** Rutgers University, New Brunswick, New Jersey, United States; Grotta Fund for Older Adults, Whippany, New Jersey, United States; The Henry and Marilyn Taub Foundation, Teaneck, New Jersey, United States; New Jersey Advocates for Aging Well, Hamilton, New Jersey, United States; Rutgers University, New Brunswick, New Jersey, United States

## Abstract

The number of communities in New Jersey formally committed to age-friendly progress grew from two in 2015 to 27 in 2024. This paper will present a case description of how engaged research helped to spur this growth. We will describe how researchers at Rutgers University helped to catalyze a private philanthropic grantmaking program to seed the development of age-friendly community initiatives across multiple municipalities in a region of the State. The grantmakers also partnered with the researchers to conduct a developmental evaluation of the grantmaking initiative. Developmental evaluation involves studying a program model that is emergent, complex, and not fully defined, with the researchers both evaluating and contributing to the intervention’s development over time. The prolonged engagement of the researchers in the initiative leaders’ work–including both formal research methods (e.g.,periodic in-depth interviews) and informal background work (e.g., participant observations)–led to creating more regular opportunities to disseminate and translate these insights back to the funders and community leaders. These developments led to the founding of Age-Friendly North Jersey, a community of practice whereby age-friendly leaders meet regularly to work toward shared goals and learn together. Informed by this success and to expand it statewide, the New Jersey Department of Human Services, Division on Aging Services, recently launched a state-supported grantmaking program to similarly invest in age-friendly community planning. Presenters will describe how this “long arch” of engaged research across systems levels is helping to fuel and sustain state and local innovations on aging in New Jersey and beyond.

